# The Gene Guessing Game

**DOI:** 10.1002/1097-0061(20000930)17:3<218::AID-YEA37>3.0.CO;2-X

**Published:** 2000

**Authors:** Ian Dunham

**Affiliations:** The Sanger Centre Wellcome Trust Genome Campus Hinxton Cambridge CB10 1SA UK

## Abstract

A recent flurry of publications and media attention has revived interest in the question of how many genes exist in the human genome. Here, I review the estimates and use genomic sequence data from human chromosomes 21 and 22 to establish my own prediction.


**Yeast**
*Yeast* 2000; **17**: 218–224.

**Review Article**

### The gene guessing game

Ian Dunham*
*The Sanger Centre, Wellcome Trust Genome Campus, Hinxton, Cambridge CB10 1SA, UK*

**Correspondence to:
I. Dunham, The Sanger Centre, Wellcome Trust Genome Campus, Hinxton, Cambridge CB10 1SA, UK.*

### Abstract

**A recent flurry of publications and media attention has revived interest in the question of how many genes exist in the human genome. Here, I review the estimates and use genomic sequence data from human chromosomes 21 and 22 to establish my own prediction. Copyright © 2000 John Wiley & Sons, Ltd.**

**Keywords: human genome; genes; DNA sequence; chromosome 22; EST**

### Introduction

How many genes are there in the human genetic parts list? Forget the 5 year plans [19] and the press releases [18] — the end of the human genome project will come when we have an accurate answer to this question. Sure, there will be a few tens or maybe even hundreds not identified, and perhaps we won't have the full structure of every gene, but we will have a pretty good idea of how many genes there are. And it is not just for the satisfaction of knowing that we have got to the end, that this number is important. One of the purposes of genomics is to allow systematic study of all genes and their products, and to remove bias inherent in working with partial datasets. The anticipated post-genomic studies will be compromised if they do not run with (almost) the full set of human genes. While monitoring of gene expression using microarrays, for example, can now sample 10 000 or more human cDNAs, ideally the experiments should sample all the genes. The announcement of the completion of the working draft sequence of the human genome would suggest we should soon have the answer. However, a recent cluster of papers [9,17,20] and a flurry of speculation [7,21] demonstrates that we still have a considerable way to go.

### What is a gene?

In order to count genes there must be a clear definition of what constitutes a gene. Although the genes that code for ribosomal, transfer and other functional RNAs are essential to the cell, it is the protein-coding genes that concern most gene counters [4,10]. Mostly this is because the protein-coding genes must contain the bulk of the functionality and therefore interest, but in part it is also because it is thought that they might be easier to count. Of course, we all know what we mean by a protein-coding gene. However these genes have a number of methods to increase complexity from a single region of DNA, including alternative use of promoters, exons and termination sites. Add to this overlapping transcription units, somatic recombination in some of the immune recognition loci, and the existence of highly similar gene families and pseudogenes, and defining a gene suddenly becomes hard. (I will leave discussion of the effects of post-translational modifications on all this to the proteomics companies.) Given the lack of consistency of the experimental estimates below, it is best to keep the definition simple and leave detailed refinement until later.

Fields *et al.*, [10] discuss these issues and draw the conclusion that what should be counted are 'distinct transcription units, or parts of transcription units, that may be translated to generate one or a set of related amino acid sequences'. In effect, the translation of putative open reading frames in the transcripts is virtual because it would be impractical to show that all the genes were actually translated in some cell and we must rely on the central dogma for that part. This definition seems sensible and it is sufficiently linked to the concept that there is a distinct canonical DNA sequence that encompasses all the variants of a single gene to enable accurate counting. All of the experimental approaches used

Copyright © 2000 John Wiley & Sons, Ltd.

so far tacitly accept that this is what they are counting.

With this definition in mind, why are human genes hard to count? Complete genome sequences of *Escherichia coli* (4300) [5], *Saccharomyces cerevisiae* (6200) [12], *Caenorhabditis elegans* (19 000) [23] and *Drosophila* (13 600) [1] have given reliable estimates of gene number in these species. The problems with human genes really centre on the greatly expanded size of the genes compared to the model organisms, particularly in the introns, which makes modelling of genes direct from genomic sequence relatively unreliable [8]. Furthermore, at the current time the working draft sequence adds to the uncertainty because it consists of 10–20 fragments/100 kb on average, with a relatively high error rate compared to complete sequence. This makes genes difficult to assemble if they span sequence contigs and may also prevent discrimination of genes from the frequent pseudogenes. Confident identification of a gene requires supporting evidence from some form of RNA transcript (cDNA or EST), or from similarity to another gene at the protein level. Even with complete high-accuracy sequence, there will still be uncertainty as to whether all genes have been identified, chiefly because there always remains the possibility that there are some genes without significant similarity to a known gene which are expressed at very low levels, in obscure tissues or for very short periods.

It is because of these uncertainties that there is still scope for estimation of gene number by a range of approaches. Most have chosen to estimate the complexity of more readily accessible fractions of the genome which act as surrogates for genes, and then to scale the estimate to the anticipated characteristics of the whole genome. The pitfalls of these methods lie in how well the chosen fraction corresponds to true genes, and what the true gene distribution is in the whole genome. In the absence of complete sequence, this is the only game in town and it can only end when there is independent convergence of multiple approaches to a common estimate.

### The methods and the estimates

### Early approximations

It is often quoted that there are 50 000–100 000 human genes. It is not clear how this estimate became fixed in the literature. Certainly, one source is from assumptions about the total coding potential of the genome. If the genome is 3000 Mb in length and an average gene is 30 kb, then there is room for 100 000 non-overlapping genes [11]. In fact, this type of approach is not as naive as it at first appears, providing correction is made for the fact that not all human genomic DNA is used for genes. The mean genomic length of the genes annotated on the complete sequences of human chromosomes 21 and 22 is at least 28 kb [13,8]. What is missing is that only 32.4% of the two chromosomal sequences is used for these genes. Adjusting for this apparent extravagance in our genomes suggests that there might be 35 000 genes.

A second source for the textbook estimate comes from the earliest experimental approaches, which analysed the complexity of mRNA species in mammalian cells using reassociation kinetics. These data were summarized by Lewin in *Genes IV* [16] to conclude that there were probably 20 000–40 000 human genes and certainly no more than 100 000. However, by *Genes V and VI* the figure had fixed on 100 000. What never seems to have been clearly established in the reassociation studies is how many mRNA species are unique to a single cell type, and consequently how far the survey of different cells must go to effectively sample all genes. Furthermore, our current knowledge of mRNAs suggests that the reassociation method is likely to be confounded by the many alternative transcripts generated from a single transcription unit.

Since then there have been a number of attempts to count gene number, resulting in estimates ranging from 30 000 to more than 120 000. Some estimates are unpublished and based on proprietary datasets or techniques [7] and hence are impossible to critically assess. However, below I review the major published estimates and revisit calculations based on the chromosome 21 and 22 genomic sequences.

### CpG islands

CpG islands are genomic sequences approximately 1 kb in size which, unlike bulk DNA, are non-methylated and contain the dinucleotide CpG at the expected frequency, given their base content. In the rest of the genome the frequency of CpG dinucleotides is suppressed. CpG islands also have a significantly higher C + G content than bulk DNA.

Copyright © 2000 John Wiley & Sons, Ltd.

CpG islands are found at the 5′ ends of many genes in mammalian genomes, and hence can be used as gene markers. Antequera and Bird [3] physically separated potential CpG island sequences from the rest of the genome on the basis of their preferential digestion with *Hpa*II. They then quantitated the amount of DNA in the CpG island fraction and, after applying a series of correction factors, calculated that there are 45 000 CpG islands in the human genome. Since a survey of the gene sequences then available in the database suggested that 56% of genes had CpG islands, they extrapolated to 80 000 genes in man. This estimate was supported by a simultaneous analysis on the mouse genome, which showed that, although there are fewer islands in mouse, the fraction of genes with CpG islands is also lower, so that the method arrives at 80 000 genes for mouse as well.

The accuracy of this estimation depends in part on the validity of the correction factors that were used to compensate for the relative contribution of tritiated thymidine from non-CpG island and CpG island DNA to the CpG island-enriched fractions. Ewing and Green [9] question several aspects of the calculation, which may lead to over-estimation of the total number of CpG islands. Furthermore, the extrapolation from CpG island number to gene number requires that every island is associated with a gene. In the chromosome 22 sequence it is clear that some pseudogenes and repeats have sequences with CpG island-like properties and that some genes with CpG islands at the 5′ end also contain other putative islands. So it is possible that this estimate is too high. However, analysis of the DNA sequence cannot assay the methylation status of the putative islands, and it is also plausible that the experimental separation is selecting almost exclusively for gene-associated CpG islands.

As a footnote to this approach, Incyte Genomics claimed to have used a CpG island-based sequencing strategy to estimate that 53% of genes have associated CpG islands and that there are just over 75 000 CpG islands in total in the genome [7]. On the basis of these figures, they predicted that there may be more than 140 000 genes. Presumably the main caveats to this estimate are whether the DNA that is being sequenced is all unmethylated CpG islands and whether all these sequences are associated with genes. Anyway, if you remain unconvinced it is possible to see and hear this estimate at http://www.incyte.comwebcast/slides/ and http://www.incyte.com/webcast/index.html. (Author's note: Simply must remember to get sound card installed in PC so that I can hear what Randy Scott is saying.)

### EST clustering

Modern sequencing technologies have allowed an alternative way to assess the complexity of mRNA in human cells. There are now large collections of single-pass sequence reads derived from cDNA clones from a broad range of human tissues, so-called expressed sequence tags (ESTs) [2,14]. ESTs may represent different portions of a mRNA, depending on whether the cDNA library was primed from polyA or randomly, and whether the sequence comes from the 5′ or 3′ end of the clone. Furthermore, in the efforts to obtain sequences of mRNAs expressed at low levels there is redundant sampling of the same mRNAs within the EST collections. Therefore considerable effort has been spent developing computational methods to assign ESTs into clusters that might reconstitute the single mRNA species. Assessment of these clusters relative to a representative sample of genes allows a simple extrapolation to the total number of genes in the genome [24]. Three papers have applied this method to human ESTs and genes [9,10,17]. Although Fields *et al.*. [10] and Ewing and Green [9] each describe the logic slightly differently, the underlying calculation is broadly the same (Figure 1). Liang *et al.*. [17] adopted a different approach to the calculation, which necessitated estimating how many EST clusters belong to the same gene.

Fields *et al.* [10] found ESTs matching 1877 of 3483 unique coding regions, leaving 40 077 novel EST clusters, and estimated between 60 000 and 70 000 genes after correction for presumed redundancy. However, included in the EST clusters were singleton ESTs, which may have inflated the estimate. There is clear evidence that EST collections contain a number of contaminants, including genomic DNA and partially spliced forms [17] as well as less accurate sequences which may not join clusters or match the test set of genes.

Both Liang *et al.* [17] and Ewing and Green [9] carefully removed artefactual and contaminating sequences from sets of over 1 million ESTs and clustered them. They each matched their clustered ESTs with either a set of human transcript sequences or the chromosome 22 genomic sequence

Copyright © 2000 John Wiley & Sons, Ltd. 

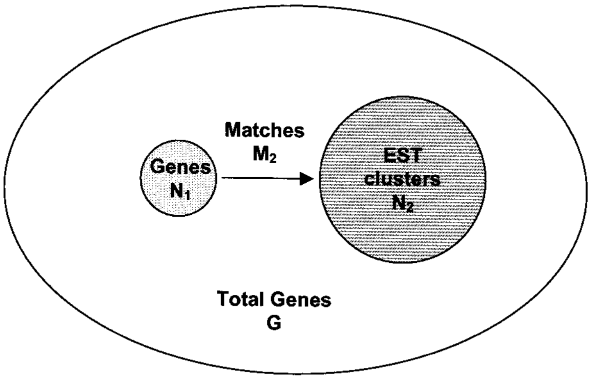

**Figure .** Estimation of gene number from two sets of gene sequences. To derive the total number of genes in the genome ($G$), take two independently derived sets of gene sequences. The first is a set of $N_1$ essentially full-length sequences of an unbiased representative sample of the genes in the genome. The second set consists of $N_2$ sequences representing genes that may be redundant and incomplete and need not be unbiased (i.e. the EST clusters). The sequence quality of the second set must be sufficient to reliably identify the matches to the sequences in the first set. The first set of genes represents some fraction, $f$, of the total number of genes given by $f = N_1/G$, and therefore should also match a corresponding fraction of the second set of sequences, $f N_2$. The experimentally determined number of sequences in the second set that match the first is $M_2$, so that $M_2 = f N_2$, and $f = M_2/N_2 = N_1/G$. Therefore the total number of genes is given by $N_1 N_2 / M_2$

to obtain estimates of 120 000 and 35 000 respectively. Why the huge difference? The discrepancy may lie in the details. The fraction of matches of the transcript test set to the clusters is different between the groups. Ewing and Green found 6169 of their 7662 mRNAs (0.805) matched EST clusters, whereas Liang *et al.*found 10 224 out of 18 665 (0.548) matching clusters. It is possible that there may be bias towards highly expressed genes in the smaller set, or the presence of transcripts which are not full-length and therefore do not match 3′ ESTs in the larger set, which might account for the difference. It is also possible that Liang *et al.* underestimated the redundancy of the EST collections because they did not compensate for the existence of EST clusters that represent the same gene but do not overlap. There are other differences too, but clearly it would be helpful to resolve the discrepancies by an exchange of data and methodologies.

### Comparative sequence analysis

Comparative sequence analysis across species offers an alternative approach. Roest Crollius *et al.* [20] used sequence representing one-third of the genome of the pufferfish, *Tetraodon nigroviridis,* to develop a similarity search tool to identify human genes. They optimized the conditions of their similarity search to detect sequence alignments between homologous pairs of pufferfish and human genes, and then applied the optimized search to compare at the translated level the *T. nigroviridis* genome sequence to test sets of human genes and cDNA sequences. This calibration demonstrated that 65–70% of test genes are detected through exon matches under conditions where no alignments fall in introns and that there are between 2.58 and 3.18 overlapping alignments of *T. nigroviridis* sequence per human gene. Applying the method to the annotated genes and pseudogenes in the human chromosome 22 sequence suggested that there were approximately 30 previously unfound genes, and that the sequence contains no more than 600 genes. 89% of the alignments within the chromosome 22 annotation were in genes while the remainder matched pseudogenes. To get to the estimate of the number of human genes, they compared the *T. nigroviridis* sequence to 42.4% of the human genome to identify 42 066 overlapping alignments. Extrapolated to the whole genome at 2.58–3.18 alignments per gene and 89% genes, this gave an estimate of 28 000–34 000 genes.

Assuming that the test sets of genes used to calibrate this method are representative, comparative sequencing of the pufferfish clearly gives a powerful tool for identification of approximately two-thirds of human genes. However, as expected, it cannot provide full gene structures as not all exons are covered. The basis for detection is probably through conserved protein domains rather than by identification of true orthologues, and hence it remains possible that with more *T. nigroviridis* sequence the one-third of human genes that could not be found will become accessible. On the other hand, there may be human genes which have little or no protein similarity with *T. nigroviridis* sequence and which can never be found.

### *Ab initio* gene prediction

Accurate identification of genes from the working draft genome sequence using the current *ab initio*

Copyright © 2000 John Wiley & Sons, Ltd.

gene prediction methods is hampered by both the nature of the working draft sequence and the false-positive rates associated with these computational approaches. In order to increase accuracy, it is possible to incorporate requirements for confirmation of gene predictions using similarity matches to proteins, ESTs or complete cDNAs [15]. The Ensembl project [22] uses this approach to provide automated annotation of the working draft sequence. On the basis of the confirmed genes found in the working draft sequence in June 2000, the Ensembl project estimated that there are 38 000 genes in the genome.

In the private sector, DoubleTwist (http://www.doubletwist.com) announced in May 2000 that they had used a similar approach of combining gene predictions with similarity searches to identify 65 000 'high confidence' genes and 40 000 other potential genes. These details were made available through the transcript of a press conference available at http://www.doubletwist.com/info/pressarticle.jsp?id = art122, but are not published. Hence, it is difficult to be sure about the reasons for the difference seen between the Ensembl and DoubleTwist estimates, which ostensibly involve similar methods. One possibility is that the higher figure represents failure to collapse predictions representing fragments of a gene present in different working draft contigs into a single gene model.

### Annotation of finished genomic sequence

The recent completion of the genomic sequence of the two smallest human autosomes offered the opportunity to assess gene content from another perspective [8,13]. Both groups provided detailed annotation of confirmed gene structures based on similarity searches to protein, EST and cDNA as well as *ab initio* gene prediction, and extrapolated to estimates of 40 000–45 000 genes in the genome. At the time of publication, for the chromosome 22 annotation there was some uncertainty about how many of the *ab initio* predictions based on Genscan would prove to be real genes, and many of the recent studies above have used the chromosome 22 data to support their observations that there are either many more genes on chromosome 22 ([17], Double Twist) or that the annotation was about right [9,20]. Since that time my group has greatly extended the initial annotation of chromosome 22, making use of new data in the sequence databases, sequence matches with *T. nigroviridis* [20] and additional screening of cDNA libraries and first-round synthesis cDNA (J. Collins, M. Goward, L. Smink, D. Beare and I. Dunham, unpublished data). From this work we have been able to annotate only 17 genes that were previously unidentified, and have demonstrated that some of the previous annotations represented multiple fragments of single genes. Furthermore, we have been able to extend many of the annotations so that an additional 440 exons (12%) have been included. Thus there are now 551 genes and 141 pseudogenes annotated on chromosome 22, excluding the variable gene segments of the immunoglobulin-$\lambda$ locus.

With these updates in mind, I have recalculated estimates of total gene number based on the chromosome 21 and 22 data individually and together (Table 1). In these calculations I treat unconfirmed gene predictions separately from annotations with similarity support. I also perform the calculations with and without compensation for the relative gene density of the two chromosomes. Taking the data for the two chromosomes together, these calculations support a figure of 30 000–39 000 genes in the genome. This estimate assumes that the combination of chromosome 21 and 22 is representative of the rest of the genome. Exactly how representative they really are remains to be seen. Although these chromosomes are gene-poor and gene-rich, respectively, they do not represent the extremes [6]. Chromosomes 17 and 19 are thought to be more gene-rich than chromosome 22, while chromosomes 4, 13, 18, and X may all be more gene-poor than chromosome 21.

### Conclusion

The most recent estimates of the total gene number for man split into two camps. In the first camp, which I call the 'gene-inflators', are estimates of 100 000 and above. In the second camp (the 'gene-deflators') are estimates below 40 000. Is it possible that simply doubling the number of genes compared to *Drosophila* or nematode can account for the additional brain and motor function on which we pride ourselves? On other hand, would it really take five times as many genes, given that great complexity could be generated by alternative splicing and post-translational modification? My assessment is

Copyright © 2000 John Wiley & Sons, Ltd. 

Table 1. Estimates of total human gene number from annotated genes on chromosome 21 and 22 genomic sequences

| Dataset | Genomic size (bp) | Genome fraction[1] | Annotated genes[2] | Annotated genes and predictions[3] | Estimated gene number[4] | Calculation[4] |
|---|---|---|---|---|---|---|
| Chr 22 | 33 400 000 | 0.0111 | 551 | | 49 500 | a |
| | | | 551 | | 36 000 | b |
| | | | 551 | | 40 500 | c |
| | | | 551 | | 28 000 | d |
| | | | | 651 | 58 500 | a |
| | | | | 651 | 42 500 | b |
| | | | | 651 | 47 500 | c |
| | | | | 651 | 33 000 | d |
| Chr 21 | 33 500 000 | 0.0112 | 193 | | 17 000 | a |
| | | | 193 | | 21 000 | b |
| | | | | 225 | 20 000 | a |
| | | | | 225 | 24 500 | b |
| Chr 21+22 | 66 900 000 | 0.0223 | 744 | | 33 500 | a |
| | | | 744 | | 30 500 | b |
| | | | | 876 | 39 000 | a |
| | | | | 876 | 35 500 | b |

[1]A genome size of 3 Gb is assumed to calculate the genome fraction.
[2]The annotated genes are either the current data for chromosome 22 or the reported genes supported by expression data for chromosome 21 [13] i.e. genes identified by *ab initio* prediction have been removed.
[3]For chromosome 22 the estimated 100 predicted gene structures which might also be genes [8] is added to the current annotated set. For chromosome 21 the supported genes plus predictions are used as described by Hattori *et al.*
[4]For calculations a, b and c total gene number is extrapolated from the number of genes annotated in the chromosome sequence to the whole genome based on the fraction of the genome represented by the chromosome sequence. The calculations are either (a) uncorrected for relative gene density on the chromosome or corrected using the factors provided by (b) Deloukas *et al.* [6], or (c) Liang *et al.* [17]. In calculation (d) the relative number of total EST clusters that match chromosome 22 sequence as given in Table 1 of Ewing and Green [9] i.e. 848/43 278 is used to extrapolate from the number of genes annotated in the sequence to the whole genome. This estimate is independent of the genome fraction represented by the chromosome 22 sequence. All total gene numbers are rounded to the nearest 500 genes.

that the larger estimates are exaggerated by the complexities of mRNA splicing and termination, the problems of collapsing multiple EST clusters into single genes, our imperfect knowledge of CpG islands, and the existence of many pseudogenes. Extended annotation of the chromosome 22 sequence has not provided any evidence that there are two or three times as many genes still to be found. In fact, very few new genes are being found and other annotations are being fused together as more complete transcript information is obtained. Unless there are many very low-copy or highly tissue-specific transcripts that are not represented in the public domain databases, I really cannot see from where 100 000 genes would come. As the genome sequence is scheduled to be completed over the next 3 years, surely we should soon have a satisfactory answer. In the meantime, if you happen to be in Cold Spring Harbor in the next year, put $5 on there being 40 000 genes for me [21].

### Acknowledgements

The author would like to thank John Collins, Charlotte Cole, Andy Mungall, David Bentley and Phil Green for their comments on the manuscript and the Wellcome Trust for financial support.

### References

1. Adams MD, Celniker SE, Holt RA, *et al.* 2000. The genome sequence of *Drosophila melanogaster*. *Science* **287**: 2185–2195.
2. Adams MD, Kelley JM, Gocayne JD, *et al.* 1991. Complementary DNA sequencing: expressed sequence tags and human genome project. *Science* **252**: 1651–1656.
3. Antequera F, Bird A. 1993. Number of CpG islands and genes in human and mouse. *Proc Natl Acad Sci U S A* **90**: 11995–11999.
4. Birney E. Gene Sweepstake: http://www.ensembl.org/genesweep.html
5. Blattner FR, Plunkett G 3rd, Bloch CA, *et al.* 1997. The complete genome sequence of *Escherichia coli* K-12. *Science* **277**: 1453–1474.

Copyright © 2000 John Wiley & Sons, Ltd.

6. Deloukas P, Schuler GD, Gyapay G, *et al*. 1998. A physical map of 30 000 human genes. *Science* **282**: 744–746.
7. Dickson D. 1999. Gene estimate rises as US and UK discuss freedom of access. *Nature* **401**: 311.
8. Dunham I, Hunt AR, Collins JE, *et al*. 1999. The DNA sequence of human chromosome 22. *Nature* **402**: 489–495.
9. Ewing B, Green P. 2000. Analysis of expressed sequence tags indicates 35,000 human genes. *Nature Genet* **25**: 232–234.
10. Fields C, Adams MD, White O, Venter JC. 1994. How many genes in the human genome? *Nature Genet* **7**: 345–346.
11. Gilbert W. 1992. *The Code of Codes*. Harvard University Press: Cambridge, MA; 83–97.
12. Goffeau A, *et al*. 1997. The Yeast Genome Directory. *Nature* **387**: 1–105.
13. Hattori M, Fujiyama A, Taylor TD, *et al*. 2000. The DNA sequence of human chromosome 21. *Nature* **405**: 311–319.
14. Hillier LD, Lennon G, Becker M *et al.* 1996. Generation and analysis of 280 000 human expressed sequence tags. *Genome Res* **6**: 807–828.
15. Kulp D, Haussler D, Reese MG, Eeckman FH. 1997. Integrating database homology in a probabilistic gene structure model. *Pac Symp Biocomput* 232–244.
16. Lewin B. Genes IV. Oxford University Press: Oxford; 466–481.
17. Liang F, Holt I, Pertea G, Karamycheva S, Salzberg SL, Quackenbush J. 2000. Gene index analysis of the human genome estimates approximately 120 000 genes. *Nature Genet* **25**: 239–240.
18. MacIlwain C. 2000. World leaders heap praise on human genome landmark. *Nature* **405**: 983–984.
19. Marshall E. 1998. NIH to produce a 'working draft' of the genome by 2001. *Science* **281**: 1774–1775.
20. Roest Crollius H, Jaillon O, Bernot A, *et al*. 2000. Estimate of human gene number provided by genome-wide analysis using *Tetraodon nigroviridis* DNA sequence. *Nature Genet* **25**: 235–238.
21. Smaglik P. 2000. Researchers take a gamble on the human genome. *Nature* **405**: 264.
22. The Ensembl Project. 2000. http://www.ensembl.org
23. The Celegans Sequencing Consortium. 1998. Genome sequence of the nematode *C. elegans*: a platform for investigating biology. *Science* **282**: 2012–2018.
24. Waterston R, Martin C, Craxton M, *et al*. 1992. A survey of expressed genes in *Caenorhabditis elegans*. *Nature Genet* **1**: 114–123.

*The*
**Genomics**
*Website at Wiley*

**www.wiley.co.uk/genomics**

The **Genomics** website at Wiley is a new and **DYNAMIC** resource for the genomics community, offering **FREE** special feature articles and new information **EACH MONTH**.

Find out more about *Comparative and Functional Genomics*, and how to view all articles published this year **FREE OF CHARGE**!

Visit the **Library** for hot books in Genomics, Bioinformatics, Molecular Genetics and more.

Click on **Primary Research** for information on all our up-to-the minute journals, including: *Genesis*, *Bioessays, Gene Function and Disease*, and the *Journal of Gene Medicine*.

Let the **Genomics** website at Wiley be your **guide** to genomics-related web sites, manufacturers and suppliers, and a calendar of conferences.

WILEY

1516

Copyright © 2000 John Wiley & Sons, Ltd. 
